# TriTrap: A Robotic Gripper Inspired by Insect Tarsal Chains

**DOI:** 10.3390/biomimetics9030142

**Published:** 2024-02-26

**Authors:** Julian Winand, Thies H. Büscher, Stanislav N. Gorb

**Affiliations:** Department of Functional Morphology and Biomechanics, Zoological Institute, University of Kiel, Am Botanischen Garten 1-9, 24118 Kiel, Germany; tbuescher@zoologie.uni-kiel.de (T.H.B.); sgorb@zoologie.uni-kiel.de (S.N.G.)

**Keywords:** morphology, tarsomeres, biomechanics, soft robot, bioinspired gripping, underactuation, biomimetics

## Abstract

Gripping, holding, and moving objects are among the main functional purposes of robots. Ever since automation first took hold in society, optimizing these functions has been of high priority, and a multitude of approaches has been taken to enable cheaper, more reliable, and more versatile gripping. Attempts are ongoing to reduce grippers’ weight, energy consumption, and production and maintenance costs while simultaneously improving their reliability, the range of eligible objects, working loads, and environmental independence. While the upper bounds of precision and flexibility have been pushed to an impressive level, the corresponding solutions are often dependent on support systems (e.g., sophisticated sensors and complex actuation machinery), advanced control paradigms (e.g., artificial intelligence and machine learning), and typically require more maintenance owed to their complexity, also increasing their cost. These factors make them unsuited for more modest applications, where moderate to semi-high performance is desired, but simplicity is required. In this paper, we attempt to highlight the potential of the tarsal chain principle on the example of a prototype biomimetic gripping device called the TriTrap gripper, inspired by the eponymous tarsal chain of insects. Insects possess a rigid exoskeleton that receives mobility due to several joints and internally attaching muscles. The tarsus (foot) itself does not contain any major intrinsic muscles but is moved by an extrinsically pulled tendon. Just like its biological counterpart, the TriTrap gripping device utilizes strongly underactuated digits that perform their function using morphological encoding and passive conformation, resulting in a gripper that is versatile, robust, and low cost. Its gripping performance was tested on a variety of everyday objects, each of which represented different size, weight, and shape categories. The TriTrap gripper was able to securely hold most of the tested objects in place while they were lifted, rotated, and transported without further optimization. These results show that the insect tarsus selected approach is viable and warrants further development, particularly in the direction of interface optimization. As such, the main goal of the TriTrap gripper, which was to showcase the tarsal chain principle as a viable approach to gripping in general, was achieved.

## 1. Biological Inspiration

**Figure 1:**
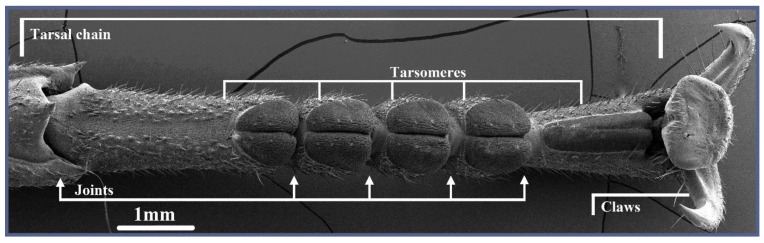
SEM micrograph of the ventral aspect of the tarsal chain in the stick insect Sungaya inexpectata. It consists of mobile-linked chain elements called tarsomeres and is strongly underactuated.

A wide variety of solutions has evolved for the locomotion and contacting/gripping of objects in living organisms [1]. Insects, in particular, show an interesting spectrum of such functions due to the presence of an exoskeleton, which provides strong variability and flexibility in evolving a multitude of various functional solutions [2,3]. The legs of some insect groups are modified to perform highly derived tasks. Some examples include praying mantises grasping prey with their forelegs [4], cicadas jumping with their hind legs [5], corbiculate bees collecting pollen with an elaborate collecting apparatus [6], webspinners using their forefeet to spin silk [7], mole crickets having shovels for digging [8], etc. As a result of evolution, a solution to a problem poses a sufficiently good compromise between energy/material investment and functional returns to be advantageous for survival [9]. Insects are often relatively short-living animals but also highly diverse in their environmental adaptations and diversity of functional solutions. They, therefore, employ a digit paradigm that, on the one hand, is comparatively simple and, on the other hand, performs well under diverse conditions with little need for direct control [10]. These properties are mainly tied to the distal part of insect legs, a strongly underactuated system called the tarsal chain (Figure 1).

These systems consist of multiple, loosely linked chain elements (tarsomeres) whose active channel of actuation is flexion along the ground-facing side of the leg [11]. In some cases, the system can also be actively bent backward [11]. This movement is facilitated by two antagonistic muscles (protractor and retractor) in spiders, but in insects, the simplest case occurs—one tendon supplemented by a few muscles differently arranged depending on the species (altogether, they represent the *musculus retractor unguis*) [12]. Additionally, the chain possesses a large number of passive degrees of freedom along other directions due to the complementary shape of the tarsomeres and their loose connection [13]. This enables the tarsus to passively conform to a large number of shapes, directions, and conditions that simple flexion of a two-segmented system would be unable to manage on its own [14,15]. Exploratory sweeping of the leg over surfaces more or less automatically predetermines a shape suitable for the task at hand. This poses a highly economical approach to contacting and thus also gripping and manipulating various objects [16]. While the chain lacks the precision and high-performance nature of dedicated and highly advanced technical manipulation devices, its relative simplicity in structure and control makes for a very good compromise between investment and returns from a performance point of view.

This attractive cost–use relationship is promising for technical systems in the fields of robotic locomotion and gripping [3]. There are ongoing efforts to push the boundaries in the development of such gripping systems, and many inspiring as well as promising approaches exist [17]. However, almost all of them either require some form of additional advanced infrastructure to work (e.g., pumps, heating, magnetic field generation, etc.), sophisticated sensory equipment (e.g., spatial/optical/force sensor arrays working in concert), and/or highly complex control mechanisms (e.g., complex adaptive movement programs, machine learning, artificial intelligence, etc.) [17]. This applies to classical “hard” robotics, where all degrees of freedom are actively controlled, as well as to the emerging field of soft robotics that attempts to employ morphological computation or encoding [18]. While this is not necessarily problematic in most scenarios, as long as these requirements result in higher performance gripping, there are some cases where this poses a hindrance. Ambient conditions, such as high humidity, high/low temperature, chemical hazards, etc., might have a detractive effect on device components of highly complex systems. Additionally, a high degree of complexity inevitably results in a higher chance of malfunction and requires more maintenance and financial investment. The insect tarsal chain is a system with a relatively simple internal working principle and, thus, few requirements with respect to construction and maintenance if replicated in a technical context [19]. Still, it provides a surprising range of possible usages, as it is seen in nature [1,2,3,4,5,6,7,8], and operates with only a few channels of direct control. In this work, an artificial gripper end effector utilizing the tarsal chain principle was designed and assembled to capitalize on these points and provide proof of concept for the tarsal chain principle.

## 2. Adopted Working Principles and Design Considerations

Tarsomeres and tarsal chains have already been used as inspiration for robotic applications, most prominently locomotion. Some recent examples include a mechanical adhesive gripper inspired by beetle claws and tarsi that was later employed in a climbing robot [20,21]; a complete walking and climbing robot called Drosiphibot modeled after the common fruit fly *Drosophila melanogaster*, which used passive tarsal chains as end effectors on its feet [22]; a spine-based wall climbing robot utilizing a tarsal chain inspired mount for its climbing spines [23]; a novel robotic gripper used in fruit harvesting that employs tarsal chain based end effectors to prevent fruit slipping after harvesting [24]; and another vertical climbing robot imitating the passive conformability of cockroach tarsal chains [25]. Another example would be the utilization of the tarsal chain structure and actuation principle in a very general sense in hyper-redundant manipulators for endoscopic surgery [26] or the creation of a tarsus-based robot leg for locomotion [27]. There have been efforts to utilize the tarsal chain principle to improve the performance of tree-climbing robots [28], to enhance the traversal of robots during the construction of orbital structures in space [29], to improve vertical traversal under outdoor conditions [30], to exploit characteristics of structures tied to tarsal chains, such as insect claws, to improve robotic gait on certain types of terrain [31], or in a climbing robot utilizing tracks inspired by tarsal chains [32]. The vast majority of work in this regard focuses on locomotion systems for robots, which is not surprising considering that tarsal chains are used for locomotion in nature as well. However, the tarsal chains’ ability to passively conform to contacted objects should lend itself well to dedicated gripping tasks on the vast majority of objects if appropriately adjusted. Therefore, it was decided here to adopt this principle and create a prototype gripping device to evaluate the tarsal chain concept and its possible implications for universal grippers.

As shown in Figure 1, the biological tarsal chain on its basic level consists of several loosely linked elements, the tarsomeres [33]. The chain’s actuation is facilitated by a tendon that runs through its whole length at a lateral offset to the pivot points between the tarsomeres. When it is pulled by its muscle, the transmitted force is exerted off-center from the joints’ neutral positions, resulting in a torque towards the tendon side of the chain. 

**Figure 2:**
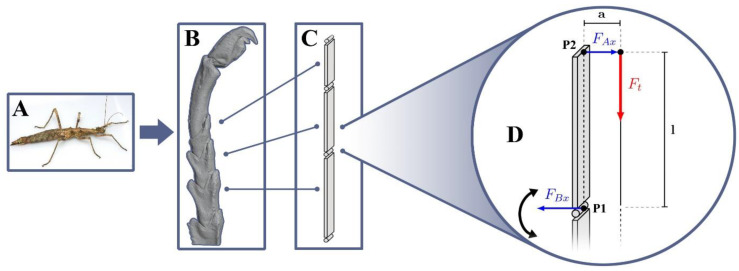
Generalized working principle of tarsal chain actuation. (**A**) Model insect species that was previously investigated, Sungaya inexpectata (distributed under the Creative Commons license (CC BY 4.0)). (**B**) A 3D model of its tarsus generated using µ-computed tomography. (**C**) The underlying, simplified mechanical setup represented as beams connected by joints. (**D**) Schematic representation of the forces at work during the actuation of individual links on the example of one tarsomere–tarsomere joint.

Figure 2 shows the generalized force interactions in such a case, using a representative, isolated element pair connected through a joint. It contains general relationships, which need to be considered in order to create an artificial, tarsal-chain-inspired folding structure. Schematically, the pulling force along the tendon *F_t_* creates a force *F_Ax_* at the tip of the upper element, which then results in a folding movement within the joint. Its magnitude can be derived from the overall reaction forces caused by the tendon pull. At the equilibrium, it holds that the sum of linearly dependent individual forces must be zero. It follows that in the case of the *x*-direction:

(1) $$\sum^{\;}F_{x}=0=F_{Ax}+F_{Bx}\;F_{Bx}=-F_{Ax}$$

Furthermore, the total equilibrium torque at point *A* must be zero:

(2) $$\sum^{\;}M\left(A\right)=0=F_{t}\cdot a+F_{Bx}\cdot l\;F_{Bx}=-\frac{F_{t}\;\cdot \;a}{l}$$

Combined with (1), it follows that the expected closing force at the tip of a given folding element can be given as

(3) $$F_{Ax}=F_{closing}=\frac{F_{t\;}\cdot \;a}{l}$$

The tendon force *F_t_* is likely limited by material and mechanical constraints in most cases [34]. Therefore, the main task for designing a biologically inspired tendon–tarsomere folding system of this kind is based on these considerations. Either the lateral offset *a* between the joint and the tendon guide needs to be sufficiently large, or the folding element length *l* needs to be small enough to permit flexion against the structure’s weight as well as expected workloads. With this criterion met, the principle can then be expanded to multiple folding elements chained together with a single tendon running through all of them along specific guides. If actuated and without further modification, the first element to fold is the topmost one because the tendon is attached there, but this order can be changed and/or reversed by introducing specifically tailored resistances to each link pair. Then, as soon as a link exhausts its angular movement space, the next one starts to fold, and so forth, until the last element has switched position, and no link is able to move anymore. 

This behavior constitutes a form of control by shape or morphological encoding [18]. Even though the only input the system receives is a linear pull at the tendon, the resulting movement is a specific sequence of different rotational and translative movements that result in a concerted, directed overall effect—it is encoded in the morphological relations of the participating elements, and not actively controlled. Furthermore, while any resistance the folding chain meets on its way to full flexion (such as an object to grip) will hinder the bending of one or more links because the tendon folds them based on the resistance force each individual link exhibits, the unobstructed links will still bend around the object that caused the obstruction. This results in good contact (conformation) between the tarsal-chain-based arm and the object without any form of active control.

This encoding can be changed by changing the structure of the device. For example, the chain tip trajectory or closing speed are functions of (a) the number of chain elements, (b) the length of chain elements, (c) the allowed maximum bending angles between elements, and (d) the order of both allowed angles and lengths along the chain (e.g., rising, falling, mixed, etc.). Additionally, as previously mentioned, the folding order could also be changed by introducing resistances of specific strengths between the chain elements, in the most basic case by mere rubber bands—which also mimics the original tarsal chain where, in many cases, relaxation is achieved by elastic reservoirs (resilin) instead of muscles [35].

Depending on how points (a) through (d) are handled, the resulting chain can potentially achieve many different actuation patterns, ranging from simple sequential folding to varying types of “embracing” movements: pulling, sideways shifting, and specific combinations of those. In this work, three identical chains of elements, called arms from now on, have been adjusted to perform embracing movements towards a common center, creating a gripping device. As described earlier in this section, these abilities of the tarsal chain are expected to lend themselves well to gripping, and the resultant gripper was supposed to prove this postulate.

## 3. Materials and Methods

The TriTrap gripper was constructed using mainly 3D-printed polylactic acid (PLA). For the computer-assisted design (CAD) process, Blender (Blender Foundation, open source) was used for all printed parts, and both the slicer programs PrusaSlicer (Prusa Research, Prague, Czech Republic) and Ultimaker Cura (Ultimaker B.V., Utrecht, The Netherlands) were used to prepare the G-code. The 3D printer was a Prusa Mk3S (Prusa Research, Prague, Czech Republic) with a 0.4 mm nozzle that used varying layer thicknesses, ranging from 0.1 to 0.3 mm, depending on the precision required for the respective parts.

Apart from 3D-printed PLA, some additional materials were used. For the tendons, a specialized fiber fishing line (Kogha Braid 12, Askari Sport GmbH, Lüdinghausen, Germany) was employed, as well as a 1.5 mm braided steel cable. The elastic returning mechanism was realized using rubber bands, which were also used to reverse the folding order of the arms to increase performance. For the assembly, 2-component epoxy glue (Plus Endfest, Uhu GmbH & Co. KG, Bühl, Germany) was used where it was not practical to print mechanical connections. In some instances, it was necessary to post-process some parts after printing for reasons of precision. For that, Proxxon (Proxxon S.A., Wecker, Germany) fine power tools were used.

**Figure 3:**
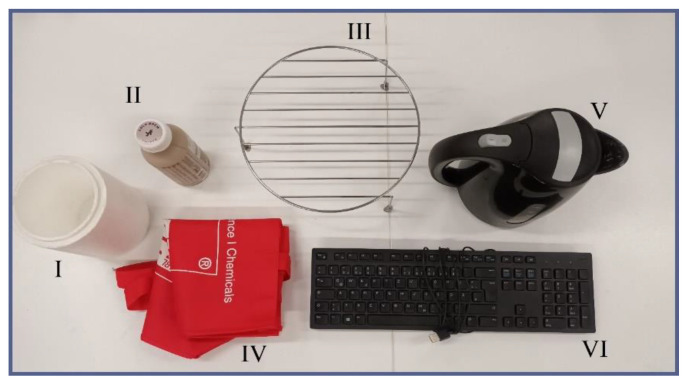
Test objects used for qualitative testing of the TriTrap gripper: I—polystyrene vase (20 g); II—empty beverage bottle made of plastic (30 g); III—steel grill attachment (160 g); IV—textile bag (40 g); V—electric water boiler (600 g); and VI—PC keyboard (400 g).

For the qualitative evaluation of the concept’s viability, a range of everyday objects with different sizes, shapes, and weights were used, as shown in Figure 3: an electric water boiler device (weight: 600 g), a PC keyboard (400 g), a textile bag (40 g), a piece of polystyrene in the shape of a vase (20 g), an empty beverage bottle made of plastic (30 g), and a grill attachment made of steel (160 g). For all objects, multiple attempts were made to pick them off the ground from the top down for two different object orientations: standing upright (where applicable) and lying on the side. While heavier objects would not be a problem for a tarsal-chain-based gripper per se, the TriTrap gripper was mainly constructed using PLA, which results in very distinct limits as to its mechanical durability. Therefore, relatively light objects were used.

## 4. The TriTrap Gripper

### 4.1. Overview

**Figure 4:**
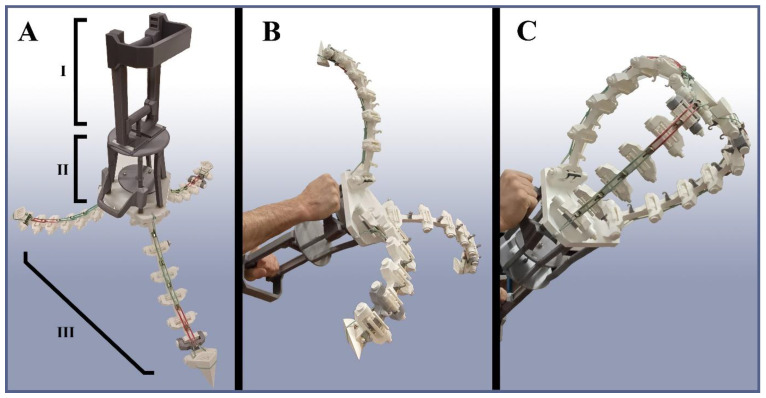
Overview of the TriTrap gripper: (**A**) open gripper, showcasing the handle (I), the actuation cage (II), and the arms (III); (**B**) gripper in hand while completely unflexed; and (**C**) gripper actuated to full flexion by hand.

The prototype gripper built in this work, called the TriTrap gripper, can be seen in Figure 4. It consists of three parts: (1) the gripper arms that perform the gripping; (2) the actuation cage, which houses the pulling handle and the force-distribution disc; and (3) the base plate, which acts as a foundation for both the arms and the cage. Each arm consists of seven linked segments, called links from now on, of which the topmost one terminates in a pointed wedge that imitates the claw found on insect legs. These wedges can be used for objects that, for reasons of shape or size, will not fit between the closed arms and provide additional support for the object inside the gripper when fully flexed. The links correspond to the tarsomeres of the biological tarsus, and rubber bands on their backsides correspond to the resilin patches found on the animal’s legs, where they act as elastic energy storage units for unfolding them again [29]. The three arms’ anchor joints are arranged in a triangle shape on the base plate, making them meet on top of it when completely flexed. The lengths of the links on each arm decrease from bottom to top the same way as in the biological model, and the allowed folding angles between the links are deliberately chosen via specifically designed angle adjusters that functionally mimic the tarsus–tarsus bending interaction in nature. This arrangement makes the arms move from their open to their closed state in an “embracing” way and causes them to meet over the center of the base plate with the faces of their wedges touching each other. In the current configuration, the TriTrap has a maximum opening angle of 90° and an open-state tip-to-tip width of 65 cm, although those specifications can be changed by changing the angle adjusters and link lengths. As for the closing speed, the arms can close as fast as actuation allows. However, the fact that there is no cushioning or braking mechanism to prevent the arms from crashing into one another with high impact necessitates caution when trying to quickly close the gripper. This issue gets compounded by the scale and, by extension, the length and mass of the arms, which naturally increases the system’s inertia. The whole device is actuated by pulling a single handle, which is situated below the base plate. The handle is connected to the arms through a force-distribution disc, which splits up the actuation force along three individual tendons associated with the arms, effectively actuating them via a single tendon per arm, as in the biological example. In its closed state, the TriTrap encloses a certain volume of space, as seen in Figure 4C. While this means that objects of sizes smaller than that cannot be properly gripped by this version of the gripper, this constitutes a design choice and not a limitation intrinsic to grippers based on tarsal chains. In the case of the TriTrap gripper, the authors intended to preserve some space between the arms to be able to equip them with different interfacing modules to increase friction in a follow-up work, which would minimize that volume in any case. 

### 4.2. Arms

**Figure 5:**
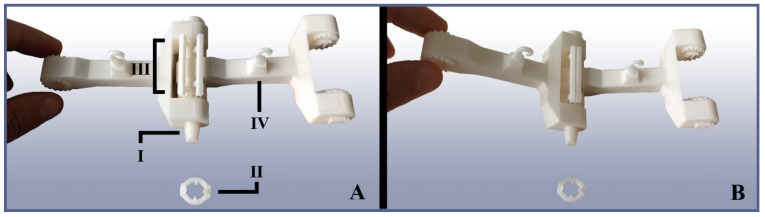
Example of the TriTrap gripper’s link–link joints. (**A**) Two isolated links connected by their joint, resting on its axle (I). The axle is held in place by a stopper piece (II), which, in this case, was detached for clarity. Any joint’s angular movement is limited by the angle adjusters (III), which can be modified to allow for different folding angles. Each link possesses a tendon guide (IV), which ensures the tendon’s relative position with respect to the joints. (**B**) The same joint in the folded state, illustrating the angle adjusters’ function.

The arms’ links are connected by monoaxial axle joints that have been designed for easy assembly and disassembly to facilitate quick replacements and part interchange, for example, to employ links of different lengths in the future. The axle runs through both connected links, as well as through an angle adjuster situated between them and terminating in a tapered head equipped with a snap-on stopper piece to hold it in place. The angle adjusters limit the maximum allowed folding angle between the links. They are designed in such a way that the angle can be changed by taking the adjuster out of the joint and rotating its cog elements relative to one another until the desired angle is locked in. Figure 5 shows an isolated link–link joint with its angle stopper and axle assembled. 

**Figure 6:**
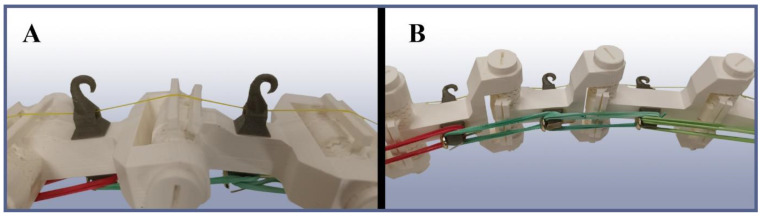
Close-up views of the arms. (**A**) Inner side of the arms, showing the tendon (yellow) and its guides that are necessary to keep it in its correct relative position with respect to the joints. (**B**) Outer side of the arms, showing the elastic relaxation (unfolding) mechanism made of standard rubber bands.

Each of the three arms is actuated into flexion by a single tendon that extends from the force-distribution disc through the base plate all the way to the arm tip, where it is attached. On its way, it runs through several specifically designed tendon guides attached to each link in order to ensure the relative position of the tendon with respect to the corresponding joint regardless of flexion state (Figure 6A). This is necessary to retain the mechanical relationships shown in Figure 2 and to keep it out of the actual gripping space. 

In the prototype, a specialist fiber fishing gear was used as the tendon. This was necessary after the mechanical demands placed upon the tendon during operation had shown themselves to exceed the properties of other, more conventional fiber types. The reversal movement, i.e., relaxation or opening, does not possess an active actuation but instead is facilitated by elastic bands on the outer side of each arm (Figure 6B). When flexing, those bands get elongated and then pull the arm back into its open state as soon as the actuation force is no longer applied. Additionally, the relative strength of the bands between different link pairs has been chosen such that the flexion movement occurs from bottom to top, as opposed to the top-to-bottom movement that would occur without elastic resistance.

### 4.3. Actuation Cage

**Figure 7:**
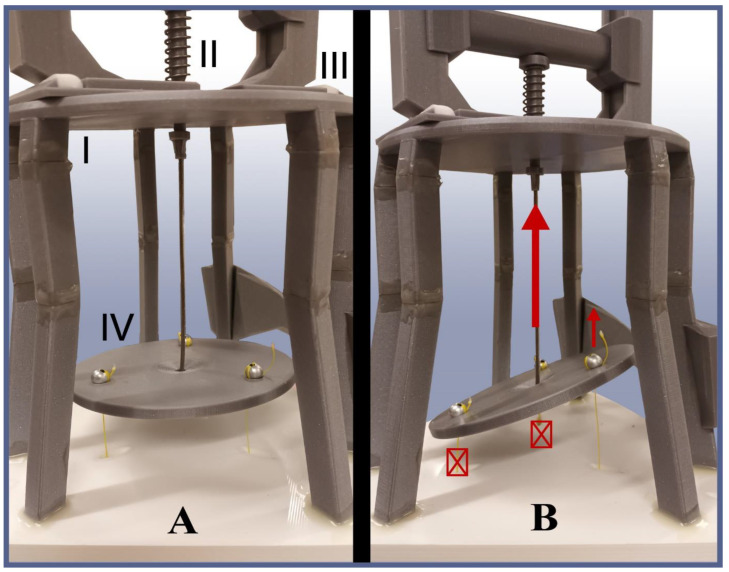
The TriTrap gripper’s actuation cage (I) and its components: tendon and preload spring (II), handle assembly (III), and force-distribution disc (IV). (**A**) Disc in equilibrium; all arms either currently require the same force to be folded or they are resting. (**B**) Tilted disc, the main actuation cord is pulled (big arrow). Two of the three arms require a stronger force to fold (crossed boxes) or might even be jammed due to the specific object’s shape. The remaining one is free to fold further (small arrow).

Below the base plate, a cage-like structure is attached, which, on the one hand, houses the force-distribution disc and, on the other hand, serves as an anchor point for the actuation handle. The force-distribution disc is a basic 3D-printed disc with four holes—one in the center and the other three at equidistant points from it—forming an equilateral triangle (Figure 7A). The three outer holes serve as attachment points for the arm tendon ends, while in the middle one, the handle cord is attached. When the handle is pulled, the handle cord exerts a pulling force at the center of the disc, which then starts to move away from the base plate. Since the arm tendons are attached to it as well, they also retract and actuate the arms they are attached to. The purpose of the disc here is twofold. First, it distributes the handle force equally among the arms, letting them flex together and in the same way as long as each of them encounters the same closing resistance. Second, in case the arms do not encounter equal closing resistance, e.g., because the object to grip possesses a strongly asymmetrical shape, it tilts to let the unjammed arms close irrespective of the jammed one (Figure 7B). This way, a single linear actuation can be used without impairing the gripper’s flexibility when it comes to adapting to the gripping object shapes.

**Figure 8:**
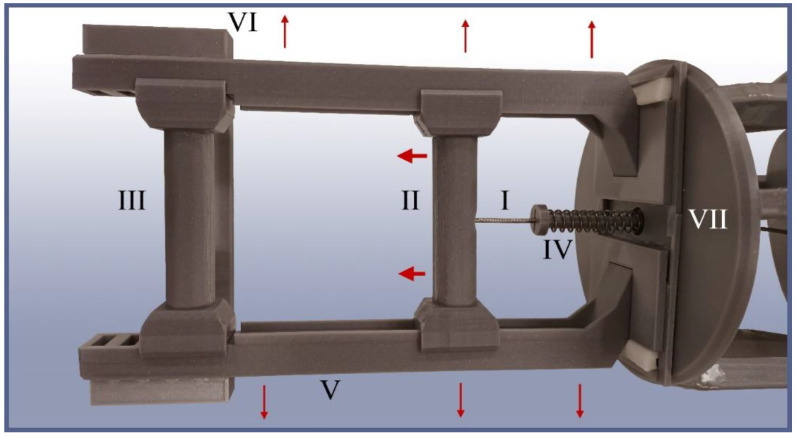
TriTrap gripper’s handle assembly. I: main actuation cord, running from the lower handle to the force-distribution disc (off to the right). II: lower handle, mounted on sliding rails to enable its movement (big arrows). III: upper handle, mounted in a fixed way as a counter grip. IV: preloader spring, used to keep a minimum strain on the system under idle conditions for handling reasons. V: sliding rails for the lower handle and overall framework for the handle assembly. VI: upper clipping lock, which keeps the sliding rails from detaching to the sides. VII: lower clipping lock, which keeps the sliding rails from detaching in the same way. If disassembly is required, the upper and lower clipping locks can be removed, and the sliding rails slide out to the sides (small arrows), granting access to the handles.

The handle consists of two bars: the fixed and the mobile one, the latter of which can be gripped and pulled at to actuate the gripper. The periphery of the handlebars, as well as the bars themselves, have been fitted with a quick disassembly mechanism that enables rapid adjustments to the travel path of the handle, if necessary, for example, in the case of different operators, different maximum closing states, or during the replacement of broken parts (Figure 8).

### 4.4. Initial Gripping Tests and Evaluation

**Figure 9:**
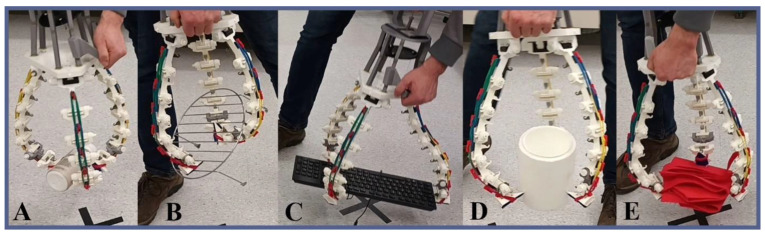
Snapshots of the TriTrap gripper gripping and transporting objects: (**A**) plastic bottle, (**B**) grill attachment, (**C**) keyboard, (**D**) polystyrene vase, and (**E**) textile bag.

After the gripper prototype had been assembled in its current working state, a range of everyday objects was used to roughly test its capabilities before moving on to optimize it (Figure 3). For each object, multiple attempts were made to pick it up, carry it, and release it again. Most objects offered more than one unique standing and/or lying state due to the absence of symmetry. Because initial tests showed that final results depended strongly on the above states chosen for the attempt, several of them were randomly picked for those objects for each gripping attempt. These results are meant to prove the concept and to highlight the direction inevitable optimization would have to go in. That being said, the gripper was able to lift all objects off the ground except for the water boiler (no success in any orientation) and managed to transport them around before dropping them off (Figure 9 and Supplementary Videos S1–S6). A common pattern observed in all gripping attempts was that success was strongly tied to the object’s surface features (i.e., rough, smooth, etc.), which was unsurprising and hinted at the necessity of a compliant and friction-increasing interface between the arms and the object. Because of this, in the TriTrap’s current state, most of the time, the wedges at the top of the arms are the elements that are actually gripping the objects instead of the arms themselves, whose passive conformability acts in a supporting fashion for the wedges.

Additionally, because the gripper was mainly constructed of 3D-printed polymers, the maximum actuation force with which the gripper could still safely be used was too low for heavier workloads, as was the case with the water boiler. 

## 5. Discussion

The TriTrap gripper presented herein has been shown to work, confirming the biomimetic concepts inspired by the insect tarsus. Even though its core principle is derived from insect tarsal chains, the gripper’s arms that are actually employing it deviate slightly in their exact construction in comparison to the biological prototype. One example would be the number of folding elements/segments corresponding to the biological tarsomeres found in the arms, which differs from the number found in most species in nature [33]. This is mainly because of geometric constraints during design and construction: a higher number (seven) was needed to fulfill some design goals, such as the arms’ ability to precisely fit each other and close in the center of the gripper (when all arms are folded), or to achieve a certain arm trajectory that was deemed advantageous for performance. 

Another deviation is the number of passive degrees of freedom: In the biological system, in addition to active flexion in the direction of the ground, the tarsal chain is able to bend along several unactuated axes at every tarsomere–tarsomere joint [11]. As mentioned in the introduction, we decided against this potentially large amount of added complexity in order to gradually approximate the working principle in a controlled fashion. Therefore, the TriTrap gripper’s link elements are monoaxial, with active actuation towards the closing direction and passive relaxation in the opposite direction. Additionally, the allowed angles between the elements were deliberately chosen for this use case, and therefore, they are not connected to those found in the biological prototype. 

In spite of all the deviations from the biological prototype and lack of optimization, the TriTrap gripper was able to prove the basic concept very well. Utilizing the tarsal chain principle found in nature, it was able to grip, hold, and move different objects without further modifications, even though there clearly is room for improvement. Firstly, the gripper’s interfaces with the objects to grip, i.e., the inner faces of the arms, are bare, hard 3D-printed PLA surfaces in the prototype. This also means that both their conformability with the gripping object, as well as the friction generation, are suboptimal. On the contrary, in the biological prototype, the tarsomeres are equipped with different interfacing structures, such as adhesive/frictional pads, spines or hairy structures, or their combinations, depending on the species [2], which alleviate this problem. In future developments of the gripper, it would clearly make sense to approximate these designs using the general design of the gripper as the platform to test various supplementary adhesive, frictional, and interlocking devices on the inner surfaces of the gripper arms. There are multiple possible ways to approach this, such as soft foam pads (corresponding to friction-generating pads found on insect legs), brushes, or other hair-type structures (corresponding to hairy structures likewise found on insect legs to improve friction and adhesion) that would be attached to the individual links. Friction- or interlocking-generating surface coatings/coverages or systems enlarging the interface area would also be possible, or a combination of these principles [2]. This is especially promising since, in some form, these types of structures are also found on the biological tarsi. Since these supplementary interfaces evolved multiple times in animal evolution [36], we can assume that they significantly contribute to gripping performance and should be replicated or approximated in the development of future technical grippers. 

Secondly, a disadvantage that made itself apparent during testing was TriTrap’s relatively unfavorable relationship between input force and output force. The tendons run through a series of channels and come into contact with different parts of the arms over their length, which generates a significant amount of friction that needs to be overcome with every actuation cycle. Additionally, the transmission ratio between tendon pull and arm flexion results in relatively high force requirements for flexion in the first place. Furthermore, for every actuation cycle, the elastic bands’ resistance needs to be overcome as well. Overall, this leads to considerable force requirements to operate the gripper, even with no target object present. Some of this is intrinsic to tarsal chains as a concept, but all of it could be significantly alleviated by further engineering and optimization. The prototype was actuated by a human hand for project simplicity, but because the required mechanical input is a simple linear pull on its tendon, a tarsal-chain-based gripper could be actuated using any other suitable means, such as a stepper motor. This also means that larger required input forces are not necessarily a problem, depending on the task and the kind of operational platform to which the gripper, as an end effector, is mounted. 

It bears mentioning that for the aforementioned reasons of force transmission and tendon friction, the gripper’s requirements with respect to mechanical material properties are relatively high. Sufficient closing forces need to be generated over the length of three multi-segmented arms. Additionally, a finalized gripper using the tarsal chain concept would need to be able to securely hold multiples of its own weight in workload, which consequently requires very durable tendon material. On the same note, the arm material itself needs to be as durable and as lightweight as possible. None of these requirements are hard to meet, but within the constraints of 3D PLA printing, which was used to produce the TriTrap gripper, there is a very distinct limit to the mechanical stability of the design. While this manufacturing method is supremely suited for rapid prototyping, in the case of industrial design, the parts should be recreated using other techniques/materials in order to reach the full potential of the gripper. 

Since the proof of concept of the insect tarsal gripper is provided here, other applications of the tarsal chain principle beyond gripping are also conceivable. Examples include but are not limited to, clamping, walking, climbing, or, with some effort, even complex manipulation. Additionally, the original gripper could be modified to tailor the gripping movement to specific applications—by increasing/decreasing the number of links on the arms and adjusting their angles, angle orders, lengths, and length orders. The potential movement variety, which could be provided by the combination of the above variables, is very large and likely contains many useful configurations. To the knowledge of the authors, not much research has yet been conducted on this topic; however, the TriTrap gripper proves to be a very promising avenue for future investigation.

## 6. Conclusions

In this study, the biological tarsal chain principle was investigated as to its viability in gripping applications. For this, a prototype biomimetic gripping device inspired by an insect tarsal chain was designed, constructed, and qualitatively tested. The device, called the TriTrap gripper, features three tarsal-chain-based arms working in concert to secure a hold of target objects. The insect tarsal chain in nature is mainly used for locomotion, featuring a relatively simple general structure and only requiring very little actuation and control to function while still being able to perform a wide range of tasks. This work tried to capitalize on these features by creating a device actuated by a single linear pulling move that nonetheless is able to adapt to varying circumstances and object shapes passively. After construction, qualitative gripping testing on a variety of objects with different shapes and sizes was carried out, which successfully proved the concept. Additionally, further avenues of optimization were determined so that the TriTrap gripper may be enhanced in the future and reach its full potential. In the end, however, the TriTrap gripper is only one of many possible examples to showcase the insect tarsal chain’s ability to passively conform to objects and features of unknown nature, which makes any gripper using the concept well suited for applications where that condition applies, such as domestic environments or different kinds of outdoor-related work, to name a few. Additionally, requiring only very basic actuation and not having to rely on advanced controls, sensors, and computation would be beneficial for many cases, as well as cheaper. 

## Data Availability

The data presented in this study are available in this article and its Supplementary Materials.

## Supplementary Materials

The following supporting information can be downloaded at: https://www.mdpi.com/article/10.3390/biomimetics9030142/s1, Video S1: Boiler; Video S2: Bottle; Video S3: Grill Attachment; Video S4: Keyboard; Video S5: Styro; Video S6: Textile Bag.
